# Evaluation of Naturally Acquired IgG Antibodies to a Chimeric and Non-Chimeric Recombinant Species of *Plasmodium vivax* Reticulocyte Binding Protein-1: Lack of Association with HLA-DRB1*/DQB1* in Malaria Exposed Individuals from the Brazilian Amazon

**DOI:** 10.1371/journal.pone.0105828

**Published:** 2014-08-22

**Authors:** Amanda Ribeiro Ferreira, Balwan Singh, Monica Cabrera-Mora, Alana Cristina Magri De Souza, Maria Teresa Queiroz Marques, Luis Cristovão Sobrino Porto, Fatima Santos, Dalma Maria Banic, J. Mauricio Calvo-Calle, Joseli Oliveira-Ferreira, Alberto Moreno, Josué Da Costa Lima-Junior

**Affiliations:** 1 Laboratory of Immunoparasitology, Oswaldo Cruz Institute, Oswaldo Cruz Foundation, (FIOCRUZ), Rio de Janeiro, RJ, Brazil; 2 Emory Vaccine Center, Yerkes National Primate Research Center, Emory University, Atlanta, Georgia, United States of America; 3 Histocompatibility and Cryopreservation Laboratory, Rio de Janeiro State University, Rio de Janeiro, Brazil; 4 National Health Foundation, Department of Entomology, Central Laboratory, Porto Velho, RO, Brazil; 5 Laboratory for Simuliidae and Onchocerciasis, Oswaldo Cruz Institute, Oswaldo Cruz Foundation, (FIOCRUZ), Rio de Janeiro, RJ, Brazil; 6 Department of Pathology, University of Massachusetts Medical School, Worcester, Massachusetts, United States of America; 7 Division of Infectious Diseases, Department of Medicine, Emory University, Atlanta, Georgia, United States of America; Centro de Pesquisa Rene Rachou/Fundação Oswaldo Cruz (Fiocruz-Minas), Brazil

## Abstract

The development of modular constructs that include antigenic regions targeted by protective immune responses is an attractive approach for subunit vaccine development. However, a main concern of using these vaccine platforms is how to preserve the antigenic identity of conformational B cell epitopes. In the present study we evaluated naturally acquired antibody responses to a chimeric protein engineered to contain a previously defined immunodominant domain of the *Plasmodium vivax* reticulocyte binding protein-1 located between amino acid positions K_435_-I_777_. The construct also includes three regions of the cognate protein (F_571_-D_587_, I_1745_-S_1786_ and L_2235_-E_2263_) predicted to contain MHC class II promiscuous T cell epitopes. Plasma samples from 253 naturally exposed individuals were tested against this chimeric protein named PvRMC-RBP1 and a control protein that includes the native sequence PvRBP1_23-751_ in comparative experiments to study the frequency of total IgG and IgG subclass reactivity. HLA-DRB1 and HLA-DQB1 allelic groups were typed by PCR-SSO to evaluate the association between major HLA class II alleles and antibody responses. We found IgG antibodies that recognized the chimeric PvRMC-RBP1 and the PvRBP1_23-751_ in 47.1% and 60% of the studied population, respectively. Moreover, the reactivity index against both proteins were comparable and associated with time of exposure (p<0.0001) and number of previous malaria episodes (p<0.005). IgG subclass profile showed a predominance of cytophilic IgG1 over other subclasses against both proteins tested. Collectively these studies suggest that the chimeric PvRMC-RBP1 protein retained antigenic determinants in the PvRBP1_435–777_ native sequence. Although 52.9% of the population did not present detectable titers of antibodies to PvRMC-RBP1, genetic restriction to this chimeric protein does not seem to occur, since no association was observed between the HLA-DRB1* or HLA-DQB1* alleles and the antibody responses. This experimental evidence strongly suggests that the identity of the conformational B cell epitopes is preserved in the chimeric protein.

## Introduction

Malaria is the most relevant parasitic disease and a leading cause of mortality in developing countries. The World Health Organization estimates that malaria was responsible for 207 million clinical cases and 627,000 deaths in 2012 [1]. The enormous progress in the implementation of malaria control measures accounts for a 45% reduction in mortality rates in the past 12 years due to their impact on *Plasmodium falciparum* malaria. These measures include long-lasting insecticidal nets (LLIN), indoor residual spraying programs (IRS) and artemisin-based combination therapy (ACT) [1]. Unfortunately, anti-vector measures do not offer protection against clinical relapses of *P. vivax* infections caused by activation of hypnozoites that occur weeks or months after primary infection. In the light of the epidemiological evidence of high morbidity, parasite drug resistance, high prevalence of severe malaria and mortality, the old concept that *P. vivax* infections are clinically “benign” is not currently accepted. It is therefore imperative to develop novel strategies for malaria control including vaccines.

The unique biological features of *P. vivax* particularly the production of hypnozoites and invasion of reticulocytes have delayed the development of experimental systems to understand parasite-host interactions. Progress toward the development of an effective vaccine has been therefore mainly focused on characterization of proteins orthologous to *P. falciparum*. Using this approach, vaccine candidates that include the pre-erythrocytic *P. vivax* circumsporozoite protein (CSP), and the sexual stage 25 kDa protein (Pvs25) have been unsuccessfully tested in clinical trials [2]–[7]. Based on the evidence that *P. vivax* in contrast to *P. falciparum* uses the Duffy binding protein (DBP) as a critical invasion ligand, this protein has been broadly studied as a vaccine candidate. DBP is localized within the merozoite's apical microneme organelles, is a member of the DBP-like erythrocyte binding protein (DBP-EBP) family and is a target of neutralizing antibodies involved in the inhibition of erythrocyte invasion [8]. Data derived from studies in endemic areas of malaria have shown that in natural conditions exposed individuals can develop broadly reactive antibodies that increase with age [9]. However, recent evidence indicates that *P. vivax* can infect Duffy blood-group negative individuals [10]. The merozoite proteins PvMSP-3 [11]–[14] and PvMSP-9 [13], [15], [16] and the apical pole protein Reticulocyte Binding Protein-1 (PvRBP1) have been also considered as potential vaccine candidates against *P. vivax* malaria.

A less characterized vaccine candidate is the *P. vivax* Reticulocyte Binding Protein-1 (PvRBP1) that forms a complex with PvRBP2 at the apical pole of the merozoite [17], [18]. It has been proposed that PvRBPs participate in a cascade of events involved in invasion by specific interaction with reticulocytes and subsequent release of the DBP essential for the junction formation step required for merozoite entry into the target host cell [19]. PvRBP1 is a relatively large Type I integral membrane protein that spans over 2,800 amino acids. The amino terminal region of the protein contains a cluster of polymorphic residues suggesting immune selection pressure [20]. The specificity of naturally acquired antibodies reactive against PvRBP1 has been reported in three different malaria exposed populations from the Brazilian Amazon [21]. High prevalence of naturally acquired antibodies against a region that spans 976 amino acids (PvRBP1_431-1407_) was reported in this study [21]. Interestingly, the fragment representing the amino terminal sequence PvRBP1_435-777_ within this fragment contains the most polymorphic region of the protein suggesting that it could be the target of functional antibodies [21]. Intriguingly, the prevalence of IgG antibodies against PvRBP1 reported by Tran et al. was lower than that observed with other *P. vivax* recombinant proteins suggesting differences in malaria transmission or differences in host population genetics. This can be consistent with the finding that *Plasmodium* polymorphic proteins are poorly immunogenic or may elicit antibody responses that are short-lived in the absence of frequent natural boosting [22]. We have reported that the genetic linkage of cognate T cell epitopes to poorly immunogenic functional domains of *Plasmodium* proteins can significantly improve their immunogenicity [23], [24]. Here we report the design of a chimeric PvRBP1 synthetic gene, codon optimized for expression in *E. coli*, that encodes a protein that includes three predicted putative promiscuous T cell epitopes, derived from different regions of the native PvRBP1 protein, arrayed in tandem and genetically fused to the immunodominant PvRBP1_435-777_ region. Since direct comparisons of the natural antibody response to these antigens provide valuable insight into how such a vaccine might work, we aimed to evaluate if the chimeric protein named PvRMC-RBP1 maintains the structural features that identified the native PvRBP1_435-777_ region as a target of naturally acquired antibodies. Comparative seroepidemiological studies were done using PvRMC-RBP1, a non-chimeric control protein PvRBP1_23-751_ and a panel of plasma samples from naturally exposed individuals with diverse HLA alleles.

## Material and Methods

### Study area and volunteers

The study involved 253 different individuals from communities in the malaria endemic region of Rondonia, a state in the western Amazon region of Brazil, where in the last five years *P. vivax* malaria accounted for more than 70% of all malaria cases. Samples and survey data were collected during the dry months of June-August of 2004 (n = 202) and 2007 (n = 51), coinciding with the period of increased malaria transmission in this region. The majority of the studied population consists of rain forest natives who have resided in the malaria-endemic region for over 25 years or transmigrants from several non-endemic areas of Brazil that have lived in Rondonia for 10 years or more. In addition, we have included as a control 30 individuals living in non-endemic regions of Brazil (Rio de Janeiro) with no history of malaria and who never resided in endemic areas. The study was reviewed and approved by the Oswaldo Cruz Foundation Ethical Committee and the National Ethical Committee of Brazil.

### Epidemiological survey

During the active case detection, subjects were informed about the forms of malaria transmission, preventive measures and the research project. Individuals who agreed to participate in our study signed an informed consent document formalizing their participation as volunteers. All volunteers were interviewed to gather personal and epidemiological data with questions related to demographics, time of residence in the endemic area, personal and family histories of malaria, use of malaria prophylaxis, presence of malaria symptoms, and personal knowledge of malaria. Survey data was entered into a database created with Epi Info 2002 (Centers for Disease Control and Prevention, Atlanta, GA).

### Human blood samples and malaria diagnosis

Blood samples (10 ml) were collected in heparinized tubes to obtain plasma used in the study. Plasma from all blood samples was separated, stored at −20°C and shipped on dry ice to the Immunoparasitology Laboratory, IOC, Fiocruz. Malaria diagnosis was made on thick and thin blood smears stained with Giemsa (Sigma Chemical Co., St. Louis, USA). Parasitemia for smear positive donors was determined by counting the number of parasites (all species and stages present) per 200 leukocytes in the thick smear. All smear-positive donors were subsequently treated for *P. vivax* or *P. falciparum* per the regimen recommended by the Brazilian Ministry of Health.

### Recombinant proteins

**Figure 1:**
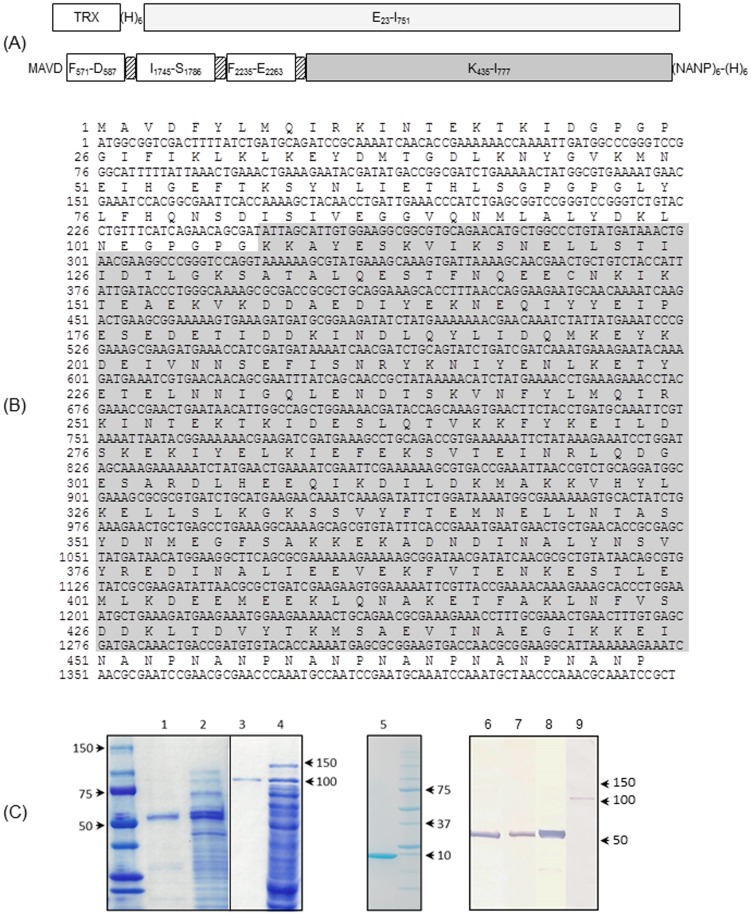
Schematic representation (A) of the recombinant proteins reported here. The control protein PvRBP1_23-751_ was expressed as an amino terminal thioredoxin (TXR)/6-histidine double fusion protein. PvRMC-RBP1 recombinant protein includes the promiscuous T cell epitopes FYLMQIRKINTEKTKID, IFIKLKLKEYDMTGDLKNYGVKMNEIHGEFTKSYNLIETHLS and LYLFHQNSDISIVEGGVQNMLALYDKLNE interspaced with GPGPG spacers and fused to the *P. vivax* RBP1 region K_435_-I_777_ Belem strain. Six copies of a *P. falciparum* sequence, derived from the repeat region of the circumsporozoite protein (NANP)_6_ were included at the C-terminus for biochemical characterization and to provide a secondary tag for protein purification. PvRMC-RBP1 also includes a C-terminal His tag that was added to the protein via the expression vector. (B) Sequence of the PvRMC-RBP1 protein. The amino acid sequence is shown in single letter code. The sequence enclosed in a gray box represents the K_435_-I_777_ fragment. The carboxyl terminal (H)_6_ tag provided by the vector was not included in the sequence. (C) SDS -PAGE of the purified PvRMC-RBP1, PvRBP1_23-751_ and thioredoxin proteins expressed in *E. coli* BL21 (DE3). The panel to the left is a composite of two gels as indicated by the solid line. Coomassie stain after SDS-PAGE separation, column purified proteins and total bacterial lysate are shown for PvRMC-RBP1 separated on 4–15% gradient gel (lanes 1 and 2) and PvRBP1_23-751_ separated on a 10% gel (lanes 3 and 4) and column purified thioredoxin separated on a 4–20% gradient gel (lane 5). The molecular weight markers (BioRad) are indicated. Western blot analysis of the purified PvRMC-RBP1 (lanes 6–8) and PvRBP1_23-751_ (lane 9) incubated with sera samples from mice immunized with a synthetic peptide representing the T cell epitope L_2235_-E_2263_ (lane 6), a synthetic peptide representing the T cell epitope I_1745_-S_1786_ (lane 7) or an anti-6X His tag monoclonal antibody (lanes 8 and 9). No reactivity was observed with sera from mice immunized with adjuvant alone or with sera from mice immunized with synthetic peptides against the control protein (data not shown).

A 1437 bp synthetic chimeric *RMC*-*Pvrbp1* gene was commercially produced by Geneart (Thermo Fisher, Regensburg, Germany) using proprietary technology platforms. The codon usage was adapted to the codon bias of *E. coli* genes using a proprietary algorithm (GeneOptimizer). The procedure was optimized to avoid AT-rich or GC-rich sequence stretches, internal TATA boxes and repeat sequences and RNA secondary structures. The optimized gene resulted in a high Codon Adaptation Index (CAI) value of 0.95 that resulted in high and stable expression rates in *E. coli* [25]. The synthetic gene encodes a chimeric protein based on the *P. vivax* RBP1 (GenBank: AAS85750.1), with the topology: MA-F_571_-D_587_-GPGPG-I_1745_-S_1786_-GPGPG-L_2235_-E_2263_GPGPG-K_435_-I_777_-(NANP)_6_ (Figure 1) designated as a PvRMC-RBP1. The protein topology is similar to that reported by us for the development of a chimeric vaccine construct based on the *P. yoelii* merozoite surface protein-1 [23]. PvRMC-RBP1 includes three putative promiscuous T cell epitopes FYLMQIRKINTEKTKID (F_571_-D_587_); IFIKLKLKEYDMTGDLKNYGVKMNEIHGEFTKSYNLIETHLS (I_1745_-S_1786_) and LYLFHQNSDISIVEGGVQNMLALYDKLNE (L_2235_-E_2263_) arrayed in tandem at the amino terminus. These regions were predicted to contain promiscuous binding peptide sequences that can bind to several HLA class II alleles as described below. Synthetic peptides were used to validate predicted epitopes as target of T cell recognition (manuscript in preparation). Validated T cell epitopes were then genetically linked to the *P. vivax* RBP1 region K_435_-I_777_ Belem strain that overlaps a previously described immune-dominant fragment [21]. GPGPG spacers were inserted between the individual promiscuous T cell epitopes and between L_2235_-E_2263_ and the native sequence K_435_-I_777_. Six copies of a *P. falciparum* sequence, derived from the repeat region of the circumsporozoite protein (NANP)_6_, were included at the carboxyl terminal end for biochemical characterization of antigenic integrity and to provide an optional affinity purification tag. The chimeric *Pvrmp1* was excised with NcoI and SacI restriction enzymes and ligated into a linearized C-terminal His tag expression vector (pET24d(+), Novagen).

A control recombinant PvRBP1 protein, with a C-terminal region that overlaps the immunodominant domain E23-V751 was generated by PCR amplification using the *P. vivax* Belem strain as a template [21] and the following primers: forward 5′GG*GAATTC*AGAAAATGCAGAAGAAGACATAAGGA3′ and reverse 5′CCAC*GAATTC*TTCTATAATTTAGCAAAAGTTTCTTTGGC3′ containing *Eco*RI restriction sites (underlined). The high fidelity thermostable KOD *Thermococcus kodakaraensis* KOD1 DNA polymerase system [26] was utilized following the manufacturer's instructions (Novagen, WI). The insert was confirmed by nucleotide sequencing. The PCR product, kindly provided by Mary Galinski (Emory University), was subcloned into pET32b (Novagen) for expression as amino terminal thioredoxin/6-His double fusion protein. Expression of the PvRMC-RBP1 and the non-chimeric PvRBP1_23–751_ was performed as described [21], [23] by re-transforming the positive clones into *E. coli* BL21 (DE3) cells (Novagen, Madison, WI) with kanamycin selection. Protein expression was induced with 1 mM IPTG for 3 hours, following standard procedures. The recombinant proteins were purified with a Ni-NTA affinity column according to the manufacturer's instructions (Qiagen, Valencia, CA). Thioredoxin was expressed and purified using similar procedures and used to determine the background antibody reactivity of the fusion protein.

To characterize the PvRMC-RBP1 protein, groups of five female BALB/c mice (8–10 weeks of age; Charles River, Wilmington, MA) were immunized with synthetic peptides representing the T cell epitopes I_1745_-S_1786_ and L_2235_-E_2263_. The mice were immunized subcutaneously three times 20 days apart with 50 µg of the individual peptides emulsified in Montanide ISA 51 (Seppic Inc., Fairfield, NJ) as described [27]. After the third immunization sera samples were collected and used for western blot analysis (Figure 1C).

### Prediction of T cell epitopes

PvRBP-1 regions containing peptide sequences promiscuous for binding to HLA class II molecules were initially predicted by using the ProPred algorithm [28]. The IEDB server (http://www.iedb.org/) was used for subsequent analysis including allele binding score and population coverage [29]. Predictions were performed for: 1) Twenty two DRB1*: 01:01; 01:02; 03:01; 03:02; 04:01; 04:02; 04:03; 04:04; 04:05; 07:01; 08:01; 08:02; 09:01; 09:02; 11:01; 11:02; 12:01; 13:01; 13:02; 14:01; 15:01; 15:02. 2) Nine DQ/DP: HLA-DPA1*01/DPB1*0401; HLA-DPA1*0103/DPB1*0201; HLA-DPA1*0201/DPB1*0501; HLA-DPA1*0301/DPB1*0402; HLA-DQA1*0101/DQB1*0501; HLA-DQA1*0102/DQB1*0602; HLA-DQA1*0301/DQB1*0302; HLA-DQA1*0401/DQB1*0402; HLA-DQA1*0501/DQB1*0201. 3) Three DRB3*: HLA-DRB3*0101; HLA-DRB3*0201; HLA-DRB3*0301; 4) One HLA-DRB4*0101 and 5) One HLA-DRB5*0101. Most predictions used the consensus algorithm in which scores equal or lower than 10 are considered to bind to the MHC allele interrogated. The fraction of individuals in human populations responding to PvRMC-RBP1 was calculated by using a computational tool provided by the IEDB server (http://tools.immuneepitope.org/tools/population/) [30]. This server uses MHC allele frequencies in human populations to calculate the fraction of individuals that might respond to a given T cell epitope when presented in the context of the MHC allele investigated. Briefly, MHC class II peptide binders were predicted in the PvRMC-RBP1 protein sequence for 22, 10 and 5 major DRB1, DQ and DRB3/B5 major MHC alleles, respectively. MHC Alleles predicted to bind sequences in PvRMC-RBP1 were used as “MHC restricted allele” input and “all population groups” presented in ethnicity field for output (Table 1).

**Table 1:** Predicted population coverage rate for PvRMC-RBP1 as predicted by IEDB.

Population/Area	Coverage^a^	Average hit^b^	PC90^c^
Amerindian	98.45%	2.82	1.78
Arab	95.03%	2.21	1.23
Asian	98.39%	2.75	1.72
Australian Aborigines	95.66%	2.2	1.26
Austronesian	94.68%	2.07	1.19
Berber	98.71%	2.25	1.53
Black	99.77%	3.86	2.67
Caucasoid	99.63%	3.13	2.16
Hispanic	99.80%	3.47	2.46
Jew	97.93%	2.42	1.49
Kurd	97.12%	2.28	1.38
Melanesian	98.99%	2.8	1.97
Mestizo	99.39%	3.18	2.18
Micronesian	97.29%	2.43	1.45
Mixed	99.56%	3.22	2.23
Mulatto	96.16%	2.05	1.27
Oriental	97.17%	2.54	1.46
Persian	98.17%	2.56	1.6
Polynesian	99.33%	2.97	2.08
Siberian	99.03%	2.91	2.02
Average	98.01%	2.71	1.76
(Standard deviation)	(1.56%)	(0.48)	(0.43)

a Projected population coverage [30].

b Average number of epitope hits/HLA combinations recognized by the population.

c Minimum number of epitope hits/HLA combinations recognized by 90% of the population.

### Antibody assays

Plasma samples from study participants were screened by ELISA for the presence of naturally acquired antibodies against the recombinant proteins. Briefly, Maxisorp 96-well plates (Nunc, Rochester, NY) were coated with 200 ng of the recombinant protein. After overnight incubation at 4°C, plates were washed with PBS containing 0.05% Tween 20 (PBS-Tween) and blocked with PBS-Tween containing 5% non-fat dry milk (PBS-Tween-M) for 1 hour at 37°C. Individual plasma samples diluted 1∶100 in PBS-Tween-M were added in duplicate wells and the plates incubated at 37°C for 1 h. After four washes with PBS-Tween, peroxidase conjugated goat anti-human total IgG (Sigma St. Louis, MO) diluted by 1∶1000 was added and plates were incubated and washed as described above. Finally o-phenylenediamine and hydrogen peroxide were used to reveal bound antibodies. The absorbance was read at 492 nm using a Spectramax 250 ELISA reader (Molecular Devices, Sunnyvale, CA). To determine specific reactivity to the control protein the averaged OD value to thioredoxin alone was subtracted from the averaged OD value to the fusion protein. This procedure was not required for the chimeric PvRMC-RBP1 given that only the His tag was incorporated into C-terminus of the protein. The results for total IgG were expressed as reactivity indexes (RI) that were calculated by dividing the mean optical density of tested samples by the mean optical density plus 3 standard deviations of 5 non-exposed controls tested on each plate. Subjects were scored positive for serum IgG to a particular antigen if the RI was higher than 1. IgG subclasses were determined in individual responders by ELISA as described above where the following peroxidase conjugated monoclonal mouse anti-human antibodies were used: Mouse Anti-Human IgG1 (hinge)-HRP (clone HP6001, Southern Biotechnology), Mouse Anti-Human IgG2 (Fc)-HRP (clone HP6002, Southern Biotechnology), Mouse Anti-Human IgG3 (hinge)-HRP (clone HP6050, Southern Biotechnology), Mouse Anti-Human IgG4 (Fc)-HRP (clone HP6023, Southern Biotechnology), all diluted by 1∶1000. Subclass-specific prevalence for each antigen was determined from OD values using 3 S.D. above the appropriate mean OD of four non-exposed controls as the cut-off for positivity.

### Absorption treatment ELISA

To ensure that the naturally acquired antibodies detected in ELISA were directed to PvRMC-RBP1 and not to the (NANP)_6_ tag used for biochemical characterization, we also performed an IgG absorption ELISA protocol using a synthetic (NANP)_6_ peptide. Briefly, flat-bottom plates (NUNC, USA) were coated overnight with 5 µg/mL of the peptide (NANP)_6_. After washing and blocking steps, plasma from 62 randomly selected PvRMC-RBP1 IgG responders were added to the plates at a 1∶100 dilution and incubated for two hours. After incubation, plasma samples were transferred to plates coated with PvRMC-RBP1 (200 ng) and the ELISA was performed as previously described.

### HLA Genotyping of PBMC

Genomic DNA was isolated from whole blood drawn in EDTA by using a mixture of 5 ml buffer G2 (QIAamp DNA Blood Midi Kit; Qiagen Inc., Chatsworth, CA, USA) and 95 µl proteinase K (20 mg/ml). After incubation at 50°C for 1 h the DNA was ethanol precipitated, collected with a glass stick and transferred into distilled water. DNA concentration and quality was checked with a NanoDrop ND-1000 spectrometer (Thermo Fisher Scientific Inc., Waltham, MA, USA). Sequence-specific oligonucleotide probes (SSOPs) and Luminex xMAP technology were used to determine the HLA-DRB1 and HLA-DQB1 allelic groups of the study populations. Briefly, the system is based on probe arrays bound to color-coded plastic microspheres, and locus-specific biotinylated primers for HLA-DRB1 and HLA-DQB1 loci (LABType, One Lambda Inc, Canoga Park, CA, USA). Biotinylated amplicons were denatured to ssDNA and incubated with DNA complementary probes immobilized on fluorescent coded microspheres (beads) followed by incubation with R-phycoerythrin conjugated to streptavidin. After hybridization, the samples were analyzed using a Luminex Flow Analyzer. The HLA Visual 2.0 software (One Lambda, CA) analysis program deduces the HLA-DRB1 and HLA-DQB1 allelic groups.

### Statistical analysis

Analyses were done using Epi Info 2002 (CDC, Atlanta, GA), Prism 5.0 and Instat (GraphPad Software, San Diego, CA) according to the required statistical test. Differences in medians for the study population data were tested by non-parametric Mann–Whitney test when appropriate. Student's t test was used to compare the means of normally distributed data or normalized transformations were performed on raw data before testing by one-way ANOVA where appropriate. Differences in the proportions of the frequencies between variables were evaluated by chi-square (χ2) test. Relationships between years of residence in the endemic area and number of past malaria episodes or months since last known malaria episode were assessed with Spearman's rank correlation. Stepwise multiple linear regressions were also used to identify which independent variables (TREA, PMI and TLI) were related to the dependent variable (IgG Reactivity index). Allelic groups were grouped by DR status and data were descriptively summarized using frequencies and percentages for all categorical variables. Overall associations of immunological responses with the alleles from each HLA-DRB1* and HLA-DQB1* loci were evaluated by comparing the allele frequencies between seronegative subjects and seropositive subjects using standard contingency tables. Each person contributed two observations to the table (one for each allele). Rare alleles, defined as those with less than five occurrences among subjects, were all pooled into a category labeled “other” for analysis. To evaluate global differences in allele distribution, we performed analyses using simulation methods as implemented in the software PASW. This approach randomly generates new cell counts for contingency tables under the null hypothesis of no association, while keeping the margins of the table fixed. We used an approach that compares each allele versus all others combined, resulting in multiple 2×2 tables, and used the maximum Chi-square statistic from this series of tables as a global test statistic (bipartition). All statistical tests were two-sided and HLA analyses were conducted using the PASW software system.

## Results

### PvRBP1 recombinant proteins

In order to develop a PvRBP1 subunit vaccine candidate, we selected in this large molecular weight protein the region spanning the amino acids 435 to 777. This fragment was selected based on the following considerations: 1) naturally exposed individuals develop antibodies which preferentially recognize this extracellular domain [21]; 2) this fragment includes a cluster of polymorphic residues suggesting that could be the target of protective antibodies and 3) it has been implied to include a functionally relevant binding domain [20]. A caveat to this approach is the loss of regions that elicit T helper responses required for the induction of antibody responses. In fact, the use of a MHC peptide-binding prediction algorithm (ProPred) indicated that although the 435–777 PvRBP1 fragment includes regions recognized by most major MHC class II DR and DQ MHC alleles, this fragment excluded several regions with very high potential for the induction of T helper responses (Table 2). As shown in Table 2, the three selected sequences have mean scores of 0.2 indicating a great potential for binding to a large number of MHC alleles. Peptides with IEDB scores below 10 are considered high binders [29]. Even more important, the predicted alleles cover a large proportion of populations in malaria endemic areas (Table 1).

**Table 2:** PvRBP-1_435-777_ and the three regions selected to design PvRMC-RBP1 are predicted to contain peptide sequences that bind to multiple HLA class II molecules.

MHC	Alleles evaluated	PvRBP1 region^a^	Alleles with predicted < = 10 score^b^	Geomean score	Min score	Max score
DRB1	22	K435-I777	22	4.4	0.1	10.0
		F_571_-D_587_	19	0.2	2.4	8.8
		I_1745_-S_1786_	17	0.2	3.1	10.0
		L_2235_-E_2263_	16	0.2	4.8	9.9
DQ	10	K435-I777	9	3.4	0.1	9.9
		F_571_-D_587_	2	6.6	4.9	8.8
		I_1745_-S_1786_	3	8.2	6.0	9.9
		L_2235_-E_2263_	8	7.5	6.0	9.7
DRB3/B5	5	K435-I777	5	2.7	0.0	9.9
		F_571_-D_587_	4	6.1	1.6	9.4
		I_1745_-S_1786_	1	4.2	2.2	6.6
		L_2235_-E_2263_	4	0.7	4.8	9.1

a The topology of the chimeric protein is summarized in Figure 1.

b Score generated by the IEDB server (http://www.iedb.org/ and [29]).

The synthetic gene encoding the PvRMC-RBP1 was codon optimized for expression in *E. coli*. The gene encoding the PvRBP1_23-751_ control protein was produced by PCR amplification and overlaps the functional domain K_435_-I_777_ included in PvRMC-RBP1 (Figure 1). PvRMC-RBP1, PvRBP1_23-751_ and thioredoxin were purified from *E. coli* lysates by metal chelate chromatography using a Ni-NTA resin. Analyses by SDS-PAGE showed that purified proteins migrated as single bands of the apparent mobility of 55 kDa for PvRMC-RBP1 and 100 kDa for PvRBP1_23-751_ (Figure 1). Biochemical identity of the recombinant proteins was established by western blot analysis using polyclonal antibodies recognizing the T cell epitopes I_1745_-S_1786_ and L_2235_-E_2263_ and a His-tag monoclonal antibody (Figure 1C).

### Epidemiological and demographical data

In this population the majority were adults and all individuals were exposed to malaria infection (Table 3). The age range was 10–85 years with an average of 35 years and the proportion of men was significantly higher (59.3%) than for women (40.7%; χ^2^ = 17∶46, p<0.0001). Concerning prior history of malaria infections, 15.9% of all studied individuals did not have or remember previous malaria infections. Among those who reported previous infections, the majority (50.8%) reported previous episodes of *P. falciparum* and *P. vivax* malaria, the two most prevalent species in Brazil [31]. The number of past infections reported by individuals varied greatly among donors, ranging from 0 to 51 (mean  = 6.74±7.58). Finally, the fact that the time elapsed since the last infection varied from 0 to 372 months (mean  = 156±312) indicated that the studied population have different degrees of exposure and/or immunity.

**Table 3:** Summary of the epidemiological characteristics of studied individuals enrolled in the survey.

Epidemiological characteristics			
Gender	N (%)	*X* ^2^	*P*
Female	103 (40. 7%)	17.46	p<0,0001
Male	150 (59. 3%)		
Total	253 (100%)		
Age (Mean ± SD)	35±16,9		
**Malaria exposure**	**(Mean±SD)**		
Time of residence in malaria endemic area	30±16.5		
Time of residence in Rondonia	24±15.2		
Time of residence in current Address	8±10		
Number of past malaria infections	6.7±7.58		
Past months since the last malaria infection	156±312		
**Previous malaria species contracted**	**N (%)**		
*P.falciparum*	28 (11. 0%)		
*P. vivax*	56 (22. 1%)		
Both species	128 (50. 5%)		
Never infected/Not reported	41 (16. 2%)		
**Species of the last infection**	**N (%)**		
*P. falciparum*	67 (26. 4%)		
*P. vivax*	114 (45. 0%)		
*P. falciparum + P. vivax*	11 (4. 3%)		
Never infected/Not reported	61 (24. 1%)		
**Diagnosis**	**N (%)**		
*P. falciparum*	07 (2.7%)		
*P.vivax*	18 (7.11%)		
*P.falciparum+P.vivax*	0 (0%)		
Not infected	228 (90.1%)		

### Frequency and magnitude of IgG immune response against recombinant antigens

**Figure 2:**
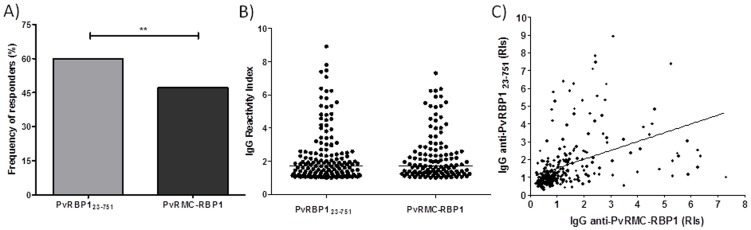
Naturally acquired IgG antibodies against PvRMC-RBP1 and PvRBP1_23-751_ recombinant proteins. (A) Frequency of IgG responders in the studied population to the recombinant chimeric protein PvRMC-RBP1 and non-chimeric PvRBP1_23-751_. The Chi squared test for proportions analyses was performed to determine statistical differences. The frequency of IgG responders to PvRBP1_23-751_ was significantly higher when compared to PvRMC-RBP1. (B) The median of the IgG reactivity index against both antigens was not significant (Mann Whitney test, p = 0.6833). (C) Reactivity indexes of IgG antibodies against PvRMC-RBP1 and PvRBP1_23-751_ showed significant correlation by nonparametric Spearman test (r = 0.5768; p<0.0001).

**Figure 3:**
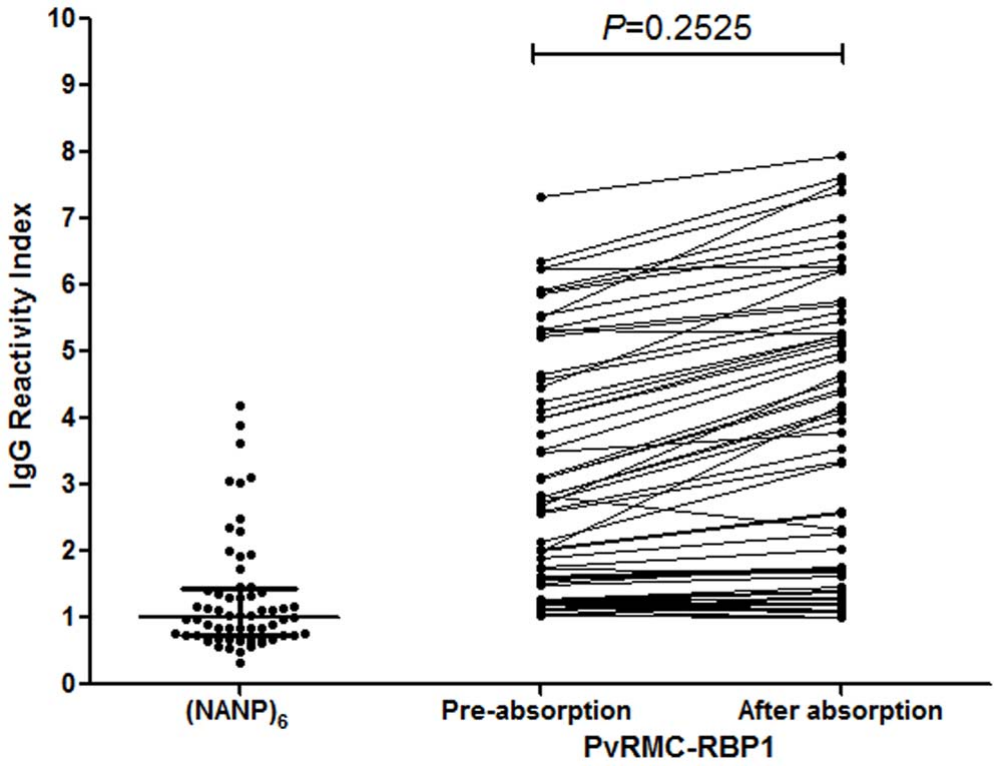
Magnitude of IgG immune response against PvRMC-RBP1 before and after (NANP)_6_-specific antibody absorption step. The median of reactivity index of IgG to PvRMC-RBP1 presented no significant difference between before and after absorption (p = 0.2525).

Assessing the humoral immune response of all 253 studied individuals against PvRMC-RBP1 and the control PvRBP1_23-751_ protein, we observed that both proteins were recognized by naturally acquired antibodies (Figure 2A). Total IgG responses were observed in 47.1% of the population to the chimeric PvRMC-RBP1 and in 60% to the non-chimeric protein PvRBP1_23-751_. There was a significant difference in the response to these two recombinant proteins (p = 0.0031). The majority of responders to PvRMC-RBP1 (83.8%) were also responders to PvRBP1_23-751_. Concerning the magnitude of the response against the PvRMC-RBP1 and PvRBP1_23-751_, the reactivity indexes (RI) of IgG antibodies ranged from 0.21 to 7.30 and did not present significant differences in responders (Figure 2B). Moreover, we also observed a significant correlation between the RIs against PvRMC-RBP1 and PvRBP1_23-751_ (Figure 2C). Interestingly, 22 individuals were responders to the PvRMC-RBP1 and were not responders to the non-chimeric PvRBP1_23-751_ (Table 4). Therefore, in order to determine if the (NANP)_6_ tag sequence plays a role in the IgG antibody reactivity against PvRMC-RBP1, we performed absorption ELISA experiments. Positive samples from 63 randomly selected individuals were pre-incubated with (NANP)_6_ synthetic peptide prior the evaluation of IgG reactivity against PvRMC-RBP1. These experiments showed that, despite that 51% of samples were positive against (NANP)_6_ after the absorption step with such synthetic peptide most plasma samples did not differ in their reactivity to PvRMC-RBP1. Consequently these results suggest that the antibodies are specific to PvRBP1 and not to the (NANP)_6_ tag (Figure 3).

**Table 4:** Frequency and magnitude of IgG response to PvRMC-RBP1 and/or PvRBP1_23-751_.

	Frequency		RI (Mean ± SD)	
	n	%	PvRMC-RBP1	PvRBP1_23-751_
PvRMC-RBP1 (+) and PvRBP1_23-751_ (+)	98	56.0%*	1.96±1.45	2.03±1.97
PvRMC-RBP1 (+) and PvRBP1_23-751_ (−)	22	12.6%	1.36±0.53	0.79±0.15
PvRMC-RBP1 (−) and PvRBP1_23-751_ (+)	55	31.4%	0.66±0.18	1.27±1.03
PvRMC-RBP1 (+) or PvRBP1_23-751_ (+)	175	100.0%		

*Frequency of IgG double responders against proteins were significantly higher than PvRMC-RBP1 (X^2^ = 73.25; p = 0.0001) and PvRBP1_23-751_ (X^2^ = 21.47; p = 0.0001) single responders.

### IgG subclass profile of responders against PvRMC-RBP1 and PvRBP1_23-751_

**Figure 4:**
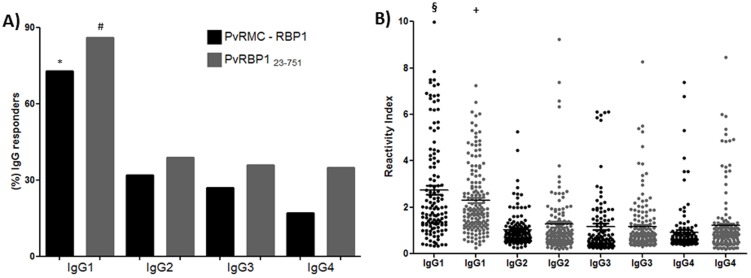
IgG subclass profile in responders against PvRMC-RBP1 and PvRBP1_23-751_ recombinant proteins. (A) Frequency of IgG subclass responders to the recombinant chimeric protein PvRMC-RBP1 and non-chimeric PvRBP1_23-751_ among the IgG responders. The Chi squared test for proportions analyses was performed to determine that the frequency of IgG1 responders was significantly higher when compared with all others IgG subclasses. (B) The medians of reactivity indexes of IgG1 subclass against both antigens tested were also higher than IgG2, IgG3 and IgG4 by Mann Whitney test. * The frequency of IgG1 responders to PvRMC-RBP1 was significantly higher when compared with IgG2 (X^2^ = 90.4; p = 0.0019), IgG3 (X^2^ = 98.6; p<0.0001) and IgG4 (X^2^ = 108.5; p<0.0001). # The frequency of IgG1 responders to PvRBP1_23-751_ was significantly higher when compared with IgG2 (X^2^ = 73.6; p<0.0001), IgG3 (X^2^ = 54.6; p<0.0001) and IgG4 (X^2^ = 55.9; p<0.0001). § The median of reactivity index of IgG1 to PvRMC-RBP1 was significantly higher than IgG2 (p<0.0001), IgG3 (p<0.0001) and IgG4 (p<0.0001) by Mann Whitney test. + The median of reactivity index of IgG1 to PvRBP1_23-751_ was significantly higher than IgG2 (p<0.0001), IgG3 (p<0.0001) and IgG4 (p<0.0001) by Mann Whitney test.

We assessed the overall distribution of the IgG antibody subclass responses to PvRMC-RBP1 and PvRBP1_23-751_ proteins using different comparative analyses. Firstly, we determined subclass-specific prevalence in total IgG positive responders for both antigens (Figure 4A). The results were comparable, IgG1 response was predominant against PvRMC-RBP1 (73.3%) and PvRBP1_23-751_ (86%) when compared respectively to IgG2 (33% and 39%), IgG3 (28% and 36%) and IgG4 (17% and 35%). Secondly, in relation to magnitude of antigen-specific IgG subclasses, we also observed that the RI of IgG1 cytophilic antibodies against PvRMC-RBP1 (1.83) and PvRBP1_23-751_ (1.95) was also significantly higher (p<0.0001) than all other subclasses (Figure 4B). We did not observe differences in the magnitude of the response for IgG subclasses against PvRMC-RBP1 and PvRBP1_23-751_ recombinant proteins.

### Influence of malaria exposure in immune response

**Figure 5:**
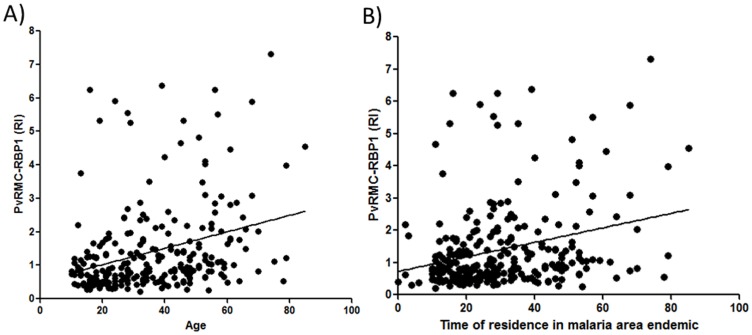
Correlation between naturally acquired IgG immune response against PvRMC-RBP1 and exposure to malaria. (A) Spearman's rank correlation between age and IgG reactivity index against the chimeric recombinant protein in a population naturally exposed to malaria (r = 0.3613; p<0.0001). (B) Spearman's rank correlation between time of residence in malaria endemic area and IgG reactivity index against the chimeric recombinant protein in a population naturally exposed to malaria (r = 0.2781; p<0.0001).

In order to assess whether epidemiological factors influence the naturally acquired immune response against PvRMC-RBP1, different parameters of the population were correlated with the reactivity indexes of total IgG and the IgG subclasses. We first observed a direct correlation between total IgG against PvRMC-RBP1 and age (r = 0.1762, p = 0.005), time of residence in endemic areas (TREA, r = 0.2781, p<0.0001) and also time of residence at the present address (TRPA, r = 0.1762, p = 0.005) (Figure 5). Assessing malaria history in relation to immune response, we also observed that the number of previous malaria infections reported also showed a direct correlation with the reactivity indexes against PvRMC-RBP1 (r = 0.1765, P = 0.0049), indicating an additive effect in specific immune response. We also used the time (months) elapsed since the last infection as indicative of protection in order to observe possible evidence of relationship with antibodies against PvRMC-RBP1 or PvRBP1_23-751_. However, we did not observe any significant correlation in the evaluation of total IgG and IgG subclasses specific against both antigens. In relation to PvRBP1_23-751_ we also observed a direct correlation between IgG reactivity indexes and exposure factors (age: r = 0.3217, p<0.0001; TREA: r = 0.2556, p<0.0001) and number of previous malaria infections (r = 0.2875, p<0.0001). Lastly, by multiple regression analysis (Table 5) we evaluated the contribution of each independent variable for the IgG magnitude against both antigens. The time of residence in an endemic area had the highest impact on acquired antibody response against the chimeric (beta = 0.294; t = 4.545; p = 0.0001) and non-chimeric (beta = 0.251; t = 3.785; p = 0.0001) PvRBP1 proteins, while time since last malaria infection was confirmed as a non-associated variable.

**Table 5:** Multivariate regression analysis of independent variables (PMI, TREA and TLI) associated with the magnitude of IgG immune response against both studied antigens.

		Unstandardized Coefficients		Standardized Coefficients		
		B	Std. Error	Beta	t	P values
PvRMC-RBP1	*constant*	0.494	0.209		2.362	0.019
	PMI	0.033	0.012	0.177	2.788	0.006
	TREA	0.025	0.005	0.294	4.545	0.0001
	TLI	0.000	0.002	−0.015	−0.227	0.821
PvRBP1_23-751_	*constant*	0.841	0.262		3.213	0.002
	PMI	0.023	0.011	0.133	2.042	0.042
	TREA	0.026	0.007	0.251	3.785	0.0001
	TLI	0.000	0.002	0.001	0.012	0.990

PMI (Past malaria infections); TREA (Time of residence in endemic area); (Time since the last malaria infection)

### HLA distribution among studied individuals and the associations with IgG response

We found 13 HLA-DRB1* and 5 HLA-DQB1* allelic groups. There were two predominant HLA allelic groups in our studied population, HLA-DRB1*04 (16% of all HLA-DR genotypes), and HLA-DQB1*03 (40% of all HLA-DQ genotypes). The HLA-DRB1*09, HLA-DRB1*10 and HLA-DRB1*12 were less frequent in HLA-DRB1* and HLA-DQB1*04 was less frequent in HLA-DQB1*. The number of positive individuals for the HLA-DRB1* and HLA-DQB1* alleles and the frequency of each allele are summarized in Table 6.

**Table 6:** Frequency (F) and number (n) of IgG responders and non-responders to the PvRMC-RBP1and Pv-RBP1_23-751_ recombinant proteins tested by HLA-DRB1* and HLA-DQB1*allelic groups from individuals naturally exposed to malaria.

HLA	F (n)	PvRMC-RBP1		Pv-RBP1_23-751_	
		Responder F	Non Responder F	Responder F	Non responder F
HLA-DRB1*					
DRB1*01	0.0988(51)	0.0737	0.1213	0.0806	0.1323
DRB1*03	0.0697(36)	0.0819	0.0588	0.0709	0.0637
DRB1*04	0.1627(84)	0.1885	0.1397	0.1774	0.1323
DRB1*07	0.0968(50)	0.0916	0.1029	0.0903	0.1078
DRB1*08	0.0910(47)	0.1024	0.0808	0.1032	0.0735
DRB1*09	0.0155(8)	0.0081	0.0220	0.0225	0.0049
DRB1*10	0.0116(6)	0.0081	0.0147	0.0096	0.0147
DRB1*11	0.1046(54)	0.1024	0.1066	0.1	0.1127
DRB1*12	0.0077(4)	0.0081	0.0033	0.0096	0.0049
DRB1*13	0.1085(56)	0.1188	0.0992	0.1064	0.1127
DRB1*14	0.0775(40)	0.0819	0.0735	0.0903	0.0588
DRB1*15	0.0794(41)	0.0737	0.0845	0.0741	0.0931
DRB1*16	0.0755(39)	0.0614	0.0882	0.0645	0.0882
HLA-DQB1*					
DQB1*02	0.1375(71)	0.1311	0.1433	0.1522	0.1421
DQB1*03	0.4031(208)	0.3934	0.4117	0.4058	0.3827
DQB1*04	0.1085(56)	0.1311	0.0882	0.1225	0.0833
DQB1*05	0.1705(88)	0.1598	0.1801	0.1612	0.1711
DQB1*06	0.1802(93)	0.1844	0.1764	0.1580	0.2205

The association between HLA-DRB1* and HLA-DQB1* alleles and haplotypes and the naturally acquired IgG response to the recombinant proteins was also evaluated. Although 30.2% of the studied individuals did not present detectable antibody titers to PvRMC-RBP1 or PvRBP1_23-751_, genetic restriction to this antigen does not seem to occur, since no association was observed between the HLA-DRB1* or HLA-DQB1* alleles and the frequency of antibody responders to both antigens (Table 6). Moreover, we also evaluated the difference between reactivity index of responders among HLA-DRB1* and HLA-DQB1* allele carriers and no difference was observed.

## Discussion

A successful malaria vaccine requires the induction of long lasting protective antibodies and robust T cell responses. However, preclinical and clinical trials have shown that a number of vaccine candidates are poorly immunogenic [32]–[35]. To overcome the poor immunogenicity of subunit vaccines, we have produced chimeric proteins that contain cognate promiscuous T helper epitopes tailored for individual antigens [23], [36]. Proof-of-principle studies in mice have shown that such chimeric vaccine constructs induced more robust antibody and T cell responses in comparison to the native protein [23], [36]. Relevantly, the magnitude and functionality of the antibody response is significantly improved using this approach [23]. Following this rationale, we predicted promiscuous T helper epitopes in PvRBP1, validated their ability to elicit T cell responses in mice (manuscript in preparation) and generated a synthetic gene encoding such promiscuous T helper epitopes genetically linked to the sequence of the PvRBP1_435-777_ domain. Interestingly, sequences of the T cell epitopes included in this chimeric protein (PvRMC-RBP1) were predicted to bind multiple HLA alleles (Table 1). According to these predictions we expect T cell responses in over 95% of the human population (Table 2). Nevertheless, chimeric proteins have the potential of generating neoantigens or masking antigenic domains that are target of protective antibodies. We report here comparative seroepidemiological studies that suggest that the identity of the conformational B cell epitopes is preserved in PvRMC-RBP1.

Seroepidemiological studies have played a significant role in the identification of leading vaccine candidates [37]–[39]. Studies using recombinant proteins representing five different regions of the PvRBP-1 protein have shown that naturally acquired antibodies preferentially recognize the amino-terminal portion of the protein that span 976 amino acids in length [21]. Interestingly, a recombinant protein containing the 317 amino acid-long amino terminal fragment of this region contains 48% of the polymorphic residues reported in the 2,749 amino acid long extracellular domain of the PvRBP-1 protein [21]. This region has also been suggested to include a functionally relevant binding domain [20]. We therefore decided to characterize here the antigenic integrity of the B cells epitopes included in the chimeric PvRMC-RBP1, in comparative experiments with a control recombinant protein that expressed the native PvRBP1_435-777_ fragment included in PvRMC-RBP1 by testing naturally acquired antibodies and their association with major HLA-DRB1* and HLA-DQB1* alleles.

Plasma samples were collected in cross-sectional studies with Brazilian Amazon communities in 2004 and 2007. The profile of studied individuals shows that our population included a majority of rainforest region natives and also transmigrants from non-endemic areas of Brazil who had lived in the area for more than 10 years. The majority of individuals reported a prior experience with *P. vivax* and/or *P. falciparum* malaria. In relation to malaria history the highly variable range of number of previous infections, time of residence in endemic area and time since the last infection suggest differences in exposure and immunity, since it is well known that the acquisition of clinical immunity mediated by antibodies depends on continued exposure to the parasite [38], [40], [41]. The correlation between time of residence in endemic areas and months since the last infection observed in our study also indicate that this phenomenon could be occurring in low/medium endemicity areas like the Brazilian Amazon. Therefore, the selection of these individuals was ideal to detect the presence of antibodies against the new recombinant antigen, distinguish whether the alterations found were related to malaria exposure and determine genetic background associated with the HLA allelic groups.

Firstly, we evaluated the naturally acquired humoral immune response mediated by total IgG antibodies against the PvRMC-RBP1 to identify the retention of naturally recognized epitopes previously reported [21]. Our results of humoral immune response mediated by IgG antibodies suggested that PvRMC-RBP1 retains the antigenic identity, being widely recognized by almost half of the studied individuals. Moreover, since *P. falciparum* infections also occur in this area, we confirmed by absorption assays that there was no significant influence of the *P. falciparum* (NANP)_6_ carboxyl terminal tag sequence in the specificity of the antibodies to PvRMC-RBP1. This frequency of responders is comparable to that reported by Tran and colleagues using five PvRBP1 fragments, including a recombinant protein representing the sequence of the fragment PvRBP1_435-777_ studied here [21]. However, the frequency of the natural antibody responses remained relatively low when compared to those against other classical *P. vivax* vaccine candidates, such as MSP-1_19_, MSP-9, MSP-3 and AMA-1, which have frequencies that ranged between 60% and 90% of reactivity against their most immunogenic regions [12], [15], [42]. On the other hand, the data is still comparable to proteins such as DBP [43] and the N-terminal region of MSP-9 [44].

In comparative experiments reported here, we observed a 13% higher prevalence of responders against the control protein compared to PvRMC-RBP1. This would indicate that structural changes resulting from the inclusion of the T-cell epitopes modified the antigenic properties of the native protein. However, our results showed that the majority of responders (82%) against PvRMC-RBP1 were also responders against the non-chimeric PvRBP1_23-751_ and the RIs were strongly correlated between both proteins, indicating that PvRMC-RBP1 preserves the antigenic domains present in the native protein that are the targets of antibody elicited by natural exposure to *P. vivax* infections. We also corroborated this hypothesis after observing the correlation with the reactivity index against the chimeric antigen with age, the time of exposure to malaria infections and the number of previous malaria infections, reflecting the cumulative effect of the specific immune response to epitopes represented in our chimeric antigen.

The absence of optimized assays to characterize functional antibodies against *P*. *vivax*, the low frequency of infected individuals and the lack of asymptomatic infections in our population, limited the evaluation of possible associations between anti-PvRMC-RBP1 antibody levels and clinical immunity. Based on the evidence that cytophilic IgG1 and IgG3 antibodies to *P. falciparum* are correlated with protection [45]–[50], whereas IgG2 and IgG4 even interfere with protective mechanisms, we also evaluated the level of reactivity and profile of IgG subclasses against both proteins. The high prevalence and magnitude of IgG1 against the chimeric and non-chimeric RBP1 recombinant proteins would indicate a protective effect. The lack of differences in frequency profile and magnitude of IgG subclasses between the studied proteins also suggest that the isotype response to the native PvRBP1 is preserved. Moreover, the high frequency of IgG1 responders observed in our work confirm previous findings with PvRBP1_431-748_, an overlapped fragment of PvRBP1_435-777_, indicating an IgG1 biased response [21]. In *P. vivax* the association between cytophilic isotypes and protection is not clearly defined. In fact, reports from our group and others already demonstrated a considerable frequency of non-cytophilic antibodies against *P. vivax* MSP-3 [51], MSP-9 [15] and MSP-1 [51] in Brazilian exposed populations.

Since many factors can contribute to the heterogeneity of the immune response to antigens and genetic restriction may influence the generation of protective immune responses to *Plasmodium* target proteins, we also aimed to investigate for the first time the association between HLA-DRB1* and HLA-DQB1* allelic groups and the immunodominant RBP-1_435-777_ fragment expressed as chimeric and non-chimeric proteins. Moreover we could investigate if the small difference in antibody response against both recombinant proteins was associated with genetic polymorphism of the HLA Class II alleles. Our results demonstrated that the studied population was heterogeneous, presenting 13 HLA-DRB1* and 5 HLA-DQB1* allelic groups. HLA-DRB1*04, HLA-DRB1*11, HLADRB1*13 and HLA-DQB1*03 were the most frequent allelic groups found in the population and the most frequent in native individuals from this Amazon area [52]. Analyses of IgG responders to PvRBP1_23-751_ and PvRMC-RBP1 showed no association between frequency and specific HLA-DRB1* and HLA-DQB1* allelic group. The lack of associations between HLA allelic groups and *P. vivax* target proteins has also been observed with other surface antigens such as PvAMA-1 and PvDBP [42]. On the other hand, in previous work with individuals from the Southwestern Brazilian Amazon, a high frequency of responders against fragments of PvMSP3-α and PvMSP-9 were defined in HLA-DRB1*04 carriers [52] while HLA-DRB1*07 was associated with the absence of antibody responses to VK210 repeats of the CSP [53]. Although computational methods for the definition of T cell epitopes is still far from perfect, these algorithms predicted a relatively large number of promiscuous T cell epitopes in PvRBP1 a finding that agrees with the lack of correlation between antibody responses to this protein and HLA types. In fact, although 40% and 52.9% of the population did not present detectable titers of antibodies to PvRBP1_23-751_ and PvRMC-RBP1 respectively, we confirmed that genetic restriction to these antigens does not seem to occur, since no association was observed between the HLA-DRB1* or HLA-DQB1* alleles and the antibody response.

Notwithstanding the naturally acquired IgG immune response against chimeric and non-chimeric PvRBP1 and the lack of HLA association with HLA-DRB1*/DQB1* reported here, it remains unknown why only a fraction of the naturally exposed individuals have antibodies against PvRBP-1. The lack of natural immune response mediated by IgG in a significant part of studied population could be explained by the presence of polymorphic residues that could be the target of antibodies [20], [21]. Moreover, since we observed a correlation with the number of previous infections, and Tran and colleagues reported a low response in recently exposed individuals from a similar area [21], it is also possible that multiple malaria episodes are necessary to induce detectable antibody titers against PvRBP1. Therefore, the presence of multiple promiscuous T cell epitopes in PvRMC-RBP1 in future immunizations could increase the humoral response against *P. vivax* Reticulocyte Binding Protein and overcome the necessity of long time exposure and infections in naturally exposed individuals.

In conclusion, our study provides valuable information concerning the chimeric PvRMC-RBP1. Firstly, the recombinant chimeric construct was broadly recognized by naturally acquired antibodies, which is correlated with time of exposure and number of malaria infections. Moreover, the predominance of the IgG1 cytophilic antibody subclass against the native and the chimeric recombinant protein also indicates a possible role in protective immunity. Lastly, our data suggest, that there was no genetic restriction mediated by HLA-DRB1* and HLA-DQB1* against this immunodominant fragment. Therefore, the confirmation that PvRMC-RBP1 has maintained its functional identity in the context of the immune response will support new studies comparing the immunogenicity in different animal models to test whether the strategy of using cognate promiscuous T cell epitopes to enhance immunogenicity can be applied for nonlinear structured domains.
